# First evidence for (TTAGG)n telomeric sequence and sex chromosome post-reduction in Coleorrhyncha (Insecta, Hemiptera)

**DOI:** 10.3897/CompCytogen.v9i4.5609

**Published:** 2015-09-02

**Authors:** Valentina G. Kuznetsova, Snejana M. Grozeva, Viktor Hartung, Boris A. Anokhin

**Affiliations:** 1 Zoological Institute, Russian Academy of Sciences, Universitetskaya nab. 1, St. Petersburg 199034, Russia; 2 Institute of Biodiversity and Ecosystem Research, Bulgarian Academy of Sciences, Blvd Tsar Osvoboditel 1, Sofia 1000, Bulgaria; 3 Museum für Naturkunde, Leibniz Institute for Evolution and Biodiversity Science, Invalidenstraße 43, 10115 Berlin, Germany; 4 Staatliches Museum für Naturkunde Karlsruhe, Erbprinzenstraße 13, 76133 Karlsruhe, Germany

**Keywords:** Karyotype, sex chromosome post-reduction, (TTAGG)*_n_* telomeric sequence, Hemiptera, Coleorrhyncha, Peloridiidae, *Peloridium pomponorum*

## Abstract

Telomeric repeats are general and significant structures of eukaryotic chromosomes. However, nothing is known about the molecular structure of telomeres in the enigmatic hemipteran suborder Coleorrhyncha (moss bugs) commonly considered as the sister group to the suborder Heteroptera (true bugs). The true bugs are known to differ from the rest of the Hemiptera in that they display an inverted sequence of sex chromosome divisions in male meiosis, the so-called sex chromosome post-reduction. To date, there has been no information about meiosis in Coleorrhyncha. Here we report a cytogenetic observation of *Peloridium pomponorum*, a representative of the single extant coleorrhynchan family Peloridiidae, using the standard chromosome staining and fluorescence *in situ* hybridization (FISH) with a (TTAGG)*_n_* telomeric probe. We show that *Peloridium pomponorum* displays 2n = 31 (30A + X) in males, the classical insect (TTAGG)*_n_* telomere organization and sex chromosome post-reduction during spermatocyte meiosis. The plesiomorphic insect-type (TTAGG)*_n_* telomeric sequence is suggested to be preserved in Coleorrhyncha and in a basal heteropteran infraorder Nepomorpha, but absent (lost) in the advanced heteropteran lineages Cimicomorpha and Pentatomomorpha. The telomere structure in other true bug infraorders is currently unknown. We consider here the inverted sequence of sex chromosome divisions as a synapomorphy of the group Coleorrhyncha + Heteroptera.

## Introduction

Coleorrhyncha (moss bugs) are little-known insects believed to be relict members (survivors) of an ancient evolutionary lineage which split off from the rest of Hemiptera during the late Palaeozoic. The suborder includes the sole extant family Peloridiidae with 17 genera and 37 species in South America (Argentina and Chile), Australia, New Zealand, New Caledonia, and Lord Howe Island. The phylogenetic relationships of peloridiids within Hemiptera, the largest nonholometabolan insect assemblage, have been a matter of contentious debates for a long time. In the past, they have been variously assigned to Heteroptera or Homoptera but today, the peloridiids are generally put to their own suborder Coleorrhyncha and are commonly considered as the sister group to the suborder Heteroptera (Wootton 1965, Schlee 1969, Wheeler et al. 1993, Campbell et al. 1995, Ouvrard et al. 2000, Burckhardt 2009, Cryan and Urban 2012), whereas there is also some support for other opinions (Cobben 1978, Popov and Shcherbakov 1996, Cui et al. 2013).

Recently, the first cytogenetic data on Coleorrhyncha were published (Grozeva et al. 2014b). Males of *Xenophyes cascus* Bergroth, 1924 from New Zealand were reported to display paired testes composed each of a single follicle, holokinetic chromosomes, a karyotype of 2n = 27 (26A + X), sex chromosome system of an X(0) type, and one chiasma in every bivalent in meiosis.

The ends of chromosomes are known to be cupped by specific nucleoprotein structures, the telomeres, which are responsible for their stability. DNA of the telomeric regions consists of tandemly repeated short nucleotide motifs. Comparative analysis of these motifs in different groups of organisms showed that they tend to be conserved in particular groups, for example, TTTAGGG in plants, TTAGGC in nematodes, TTAGG in arthropods, and TTAGGG in vertebrates (Traut et al. 2007, Lukhtanov and Kuznetsova 2010). Information on the telomere structure is presently available for many groups of insects (Sahara et al. 1999, Frydrychová et al. 2004, Vitková et al. 2005, Grozeva et al. 2010, 2011, Kuznetsova et al. 2012, 2015, Maryańska-Nadachowska et al. 2013, Golub et al. 2014, 2015, Gokhman et al. 2014, Vershinina et al. 2015, Lachowska-Cierlik et al. 2015). Among insects, the (TTAGG)*_n_* sequence has been detected in most tested orders and is considered as the ancestral telomeric motif not only for insects but also for all arthropods (Vitková et al. 2005). Despite of this, in several insect groups, e.g. in Diptera, in some Coleoptera and Hymenoptera, the classical insect-type (TTAGG)*_n_* motif is absent (Frydrychová and Marec 2002, Frydrychová et al. 2004, Vitková et al. 2005, Gokhman et al. 2014). Among Hemiptera Heteroptera, the advanced infraorders Cimicomorpha and Pentatomomorpha also appear to have lost this telomeric motif (Grozeva et al. 2011 and references therein, Golub et al. 2015). Although data on other infraorders were absent, there was a general belief that all members of the Heteroptera lost the (TTAGG)*_n_* motif (Frydrychová et al. 2004, Lukhtanov and Kuznetsova 2010, Grozeva et al. 2010, 2011). However, the recent finding of this motif in a more basal true bug infraorder Nepomorpha (Kuznetsova et al. 2012) showed that the Heteroptera are heterogeneous for the telomere organization.

The Heteroptera are known to differ from the rest of the Hemiptera in that they display an inverted sequence of sex chromosome divisions during spermatocyte meiosis, the so-called sex chromosome post-reduction. To date, there has been no information about meiosis in Coleorrhyncha.

In this paper we present first data on telomere structure and male meiosis in Coleorrhyncha. We report the karyotype, meiosis with special reference to the behavior of sex chromosomes, and molecular composition of telomeres in males of *Peloridium pomponorum* Shcherbakov, 2014.

## Material and methods

Specimens of *Peloridium pomponorum* were collected at the Biological Station Senda Darwin (Chile, Region X, Isla Grande de Chiloé, Ancud) in January-February 2014 from three different species of *Sphagnum* Linnaeus, 1753 (*Sphagnum fimbriatum* Wilson, 1847, *Sphagnum magellanicum* Bridel, 1798 and *Sphagnum falcatulum* Bescherelle, 1885) and *Hypnum chrysogaster* Müller, 1851; fixed alive in 3:1 ethanol/acetic acid and shipped in the fixative a couple of weeks later to the lab, where further analyses were undertaken.

Preparations were made from testes, which were dissected in a drop of 45% acetic acid and squashed under a coverslip on a glass microscope slide. The slides were frozen using dry ice, the coverslips were removed with a razor blade, and the preparations were air dried.

Spread chromosome plates were found in testes of 19 males (a total of 32 adults and the last instar nymphs were examined). For the standard staining, the method described in Grozeva and Nokkala (1996) with minor modifications was used. In brief, the preparations were first subjected to hydrolysis in 1 N HCl at room temperature for 20 min, then at 60 °C for 8 min and stained in Schiff’s reagent for 20 min. After rinsing thoroughly in distilled water, the preparations were additionally stained in 4% Giemsa in Sorensen’s buffer, pH 6.8 for 20 min, rinsed with distilled water, air-dried, and mounted in Entellan.

The molecular structure of telomeres was investigated by fluorescence *in situ* hybridization of chromosomes (FISH) with a (TTAGG)*_n_* probe. The telomere probe was generated by non-template PCR and labelled with Rhodamine-5-dUTP (GeneCraft, Cologne, Germany). FISH was performed as described in Grozeva et al. (2011, 2014a). Chromosome preparations were treated with 100 μg/ml RNaseA, incubated in 5 mg/ml pepsin in 0.01 M HCl to remove excessive amounts of RNAs and proteins. After pretreatment, the chromosomes were hybridized with a hybridization mixture containing about 100 ng of labelled probe and 10 μg of sonicated salmon-sperm DNA (Sigma-Aldrich, St. Louis, MO, USA).

Chromosomes were mounted in an antifade medium (ProLong Gold antifade reagent with DAPI, Invitrogen) and covered with a glass coverslip. Chromosome slides were analyzed under a Leica DM 6000 B microscope; images were taken with a Leica DFC 345 FX camera using Leica Application Suite 3.7 software with an Image Overlay module.

## Results

**Figures 1–5.**
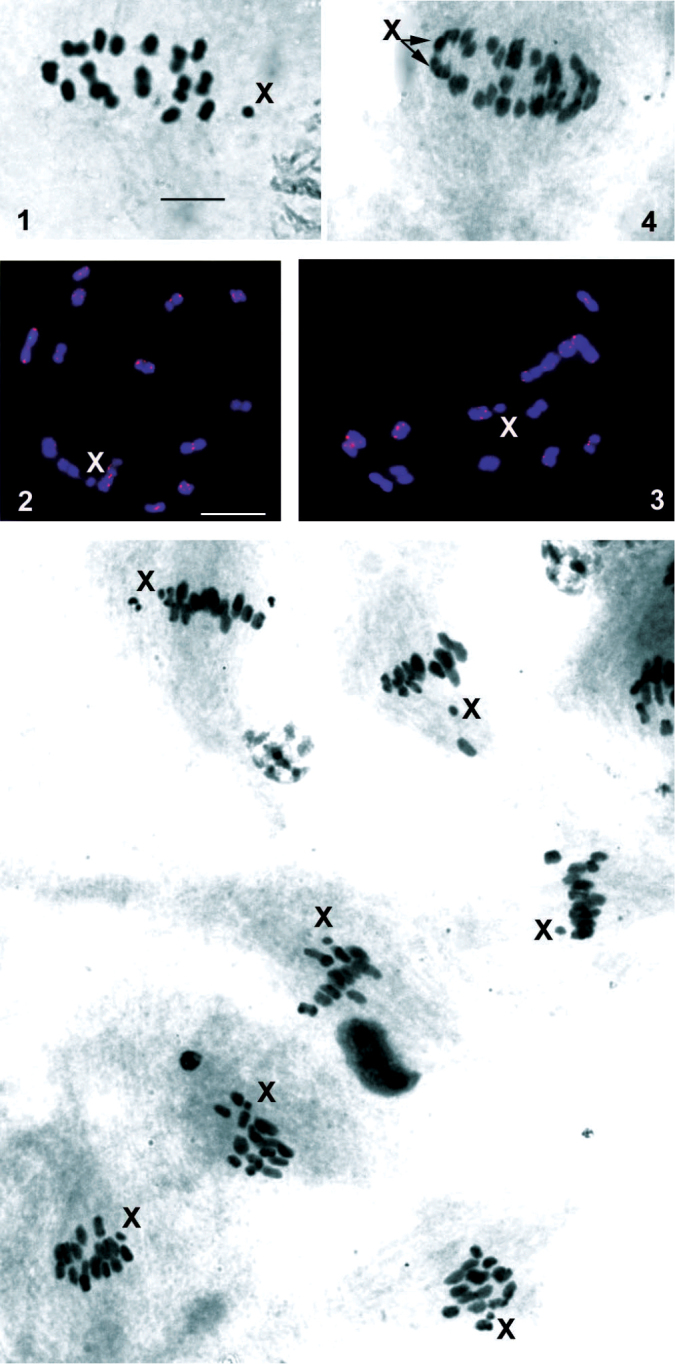
Male meiotic chromosomes of *Peloridium pomponorum* subjected to standard staining (**1, 4, 5**) and FISH with a (TTAGG)*_n_* telomeric probe (**2, 3**). **1** MI, n = 16 (15AA + X) **2, 3** MI, n = 16 (15AA + X); hybridization signals (red) are located at the ends of chromosomes **4** AI, the sister chromatids (arrowed) of X chromosome are separated and oriented toward opposite spindle poles **5** part of a secondary spermatocyte cyst; X chromosome is present in every MII plate evidencing for the equational division during the first division. Bar = 10 µm.

In *Peloridium pomponorum* males, the paired testes are composed each of a single follicle, and the meiotic karyotype comprises 16 elements, including 15 autosomal bivalents and a univalent X chromosome, at first metaphase (MI) (Figs 1–3). Thus, the male diploid karyotype of the species consists of 2n = 31 (30A + X). The chromosomes show no primary constrictions (the centromeres), thereby testifying that they are holokinetic and display, instead of localized, a diffuse kinetochore. The bivalents constitute a continuous series gradually decreasing in size and form each one, rarely two, chiasmata. The X chromosome appears as one of the smallest chromosomes in the karyotype. At the first anaphase (AI), the autosomes segregate reductionally, whereas the univalent sex chromosome undergoes the equational division (the separation of sister chromatids) (Fig. 4), so that all the second metaphases (MII) carry the X chromosome (Fig. 5). It is notable that the X chromosome tends to be situated outside the division plane both in MI and MII plates.

In all the preparations, a (TTAGG)*_n_* telomeric probe hybridized to the ends of the chromosomes (Fig. 2, 3) indicative of the presence of this telomeric nucleotide sequence in *Peloridium pomponorum*.

## Discussion

So far, the only coleorrhynchan species with known karyotype was *Xenophyes cascus* (Grozeva et al. 2014b). Males of this species originated from New Zealand were shown to have holokinetic chromosomes, as all other Hemiptera, and karyotype of 2n = 27 (26A + X). Despite difference in the number of autosomes, i.e. 26 in *Xenophyes cascus* while 30 in *Peloridium pomponorum* from Chile analyzed here, these species appear similar in that they have paired testes consisting each of a single follicle (a pattern probably shared by all peloridiids; Grozeva et al. 2014b), holokinetic chromosomes (like in all other Hemiptera; White 1973), the formation of one, rarely two, chiasmata per bivalent (a characteristic property of holokinetic bivalents; Nokkala et al. 2004), the sex chromosome system of an X(0) type, decreasing size differences between chromosomes, and the X as one of the smallest chromosomes of the set.

The male diploid chromosome number in true bugs ranges from 2n=4 to 2n=80; however, the great majority of the studied species show 2n varying between 14 and 34 (Papeschi and Bressa 2006) and, thus, both chromosome numbers found to date in peloridiid species fall into this range.

The X(0) sex determination system is generally accepted as an ancestral one in Insecta (Blackman 1995). This system is prevailing in most Hemiptera, with the only exception of the Heteroptera. In this group, an XY system appears characteristic of the overwhelming majority of studied species whereas an X(0) system occurs only sporadically, being encountered in separate representatives of both primitive and advanced taxa (Ueshima 1979, Papeschi and Bressa 2006, Kuznetsova et al. 2011). Two contradictory hypotheses for the evolution of sex chromosomes in true bugs supported by different sources of evidence have been proposed. One of these holds that the XY system has evolved from an X(0) system (Ueshima 1979) while the other assumes that the XY mechanism is plesiomorphic, the existence of the X(0) true bug species being a result of the repeated loss of the Y chromosome during the evolution (Nokkala and Nokkala 1983, 1984, Grozeva and Nokkala 1996). Deducing the ancestral state of a character for the taxon requires knowledge on this character state in the basal taxa. Of the two most primitive true bug infraorders, Enicocephalomorpha and Dipsocoromorpha (Štys and Kerzhner 1975), the cytogenetic information is currently available for the six species of the latter (Cobben 1968, Grozeva and Nokkala 1996). Species of the genera *Alpagut* Kıyak, 1995, *Cryptostemma* Herrich-Schaeffer, 1835 and *Pachycoleus* Fieber, 1860 (the family Dipsocoridae) were shown to differ both in chromosome number and sex chromosome systems. Specifically, *Alpagut castaneovitreus* (Linnavuori, 1951) displays 2n = 22 (18A + 2m? + XY) (Grozeva and Nokkala 1996, as Cryptostemma (Harpago) castaneovitreus Linnavuori, 1951); *Cryptostemma hickmani* Hill, 1987 – 2n = 22 (18A + 2m + XY); *Pachycoleus pusillimus* (J. Sahlberg, 1870) – 2n = 21 (16A + 2m + XY_1_Y_2_); while *Pachycoleus waltli* Fieber, 1860 (Cobben 1968: as *Pachycoleus rufescens* J. Sahlberg, 1875) – 2n = 21 (20A + X). On the other hand, both studied representatives of the family Schizopteridae, namely, *Pateena elimata* Hill, 1980 and *Rectilamina australis* Hill, 1984, were found to share 2n = 33 (30A + 2m + X) (Grozeva and Nokkala 1996). The occurrence of an X(0) system both in dipsocorids and schizopterids as well as in peloridiids seems to favor the Ueshima’s (1979) hypothesis, however, much more data from the primitive true bug taxa are needed to choose with certainty between the two alternatives.

With very rare exceptions (e.g. the family Tingidae; Ueshima 1979, Golub et al. 2015), true bugs show an inverted sequence of sex chromosome divisions in male meiosis, the so-called “sex chromosome post-reduction” (Ueshima 1979). It means that, in spermatocyte meiosis the first division is reductional for the autosomes and equational for the sex chromosomes, whereas the second division is, on the other hand, reductional for the sex chromosomes and equational for the autosomes. The significance of this unusual pattern is unknown. We observed that *Peloridium pomponorum* males likewise share the sex chromosome post-reduction. Taking into account that all other members of the Hemiptera display the “normal” pre-reductional sequence of sex chromosome divisions (White 1973), we consider the inverted sequence as a synapomorphy of the group Coleorrhyncha + Heteroptera.

Recently, it has been shown that the classical insect (TTAGG)*_n_* telomeric sequence is absent in members of the evolutionarily advanced true bug infraorders Cimicomorpha (at least in the families Miridae, Cimicidae and Tingidae for which data are available) and Pentatomomorpha (at least in the families Pyrrhocoridae and Pentatomidae for which data are available) (Frydrychová et al. 2004, Grozeva et al. 2010, 2011, Golub et al. 2015). Moreover, dot-blot hybridization of genomic DNA from the cimicomorphan species *Cimex lectularius* Linnaeus, 1758, *Oxycarenus lavaterae* (Fabricius, 1787), and *Nabis* sp. did not suggest any other candidate telomeric sequence, including, besides the insect TTAGG, also ciliate TTTTGGGG and TTGGGG, nematode TTAGGC, shrimp TAACC, vertebrate TTAGGG, and plant TTTAGGG (Grozeva et al. 2011) thus leaving the question of the telomeric motif(s) in these species open.

However in more recent times, the presence of the (TTAGG)*_n_* telomeric repeat was documented by FISH for the family Belostomatidae from a more basal true bug infraorder Nepomorpha (Kuznetsova et al. 2012) and now is confirmed for the peloridiid species *Peloridium pomponorum*. These new findings reinforce the hypothesis (Kuznetsova et al. 2012) that the plesiomorphic insect-type (TTAGG)*_n_* telomere structure preserved in the basal true bug taxa was subsequently lost during the further evolution of the Heteroptera, at least in the last ancestor of a monophyletic lineage including Pentatomomorpha and Cimicomorpha.

## References

[B1] Blackman RL (1995) Sex determination in insects. In: Leather SR Hardie J (Eds) Insect Reproduction. CRC Press, Boca Raton, 57–94.

[B2] Burckhardt D (2009) Taxonomy and phylogeny of the Gondwanan moss bugs or Peloridiidae (Hemiptera, Coleorrhyncha). Deutsche Entomologische Zeitschrift 56(2): 173–235. doi: 10.1002/mmnd.200900019

[B3] Campbell BC Steffen-Campbell JD Sorensen JT Gill RJ (1995) Paraphyly of Homoptera and Auchenorrhyncha inferred from 18S rDNA nucleotide sequences. Systematic Entomology 20: 175–194. doi: 10.1111/j.1365-3113.1995.tb00090.x

[B4] Cobben RH (1968) Evolutionary trends in Heteroptera. Part I. Eggs, architecture of the shell, gross embryology and eclosion. Center for Agricultural Publishing & Documentation, Wageningen, 475 pp.

[B5] Cobben RH (1978) Evolutionary Trends in Heteroptera. Part II. Mouthpart-structures and feeding strategies. Mededelingen Landbouwhogeschool, Wageningen 78(5): 1–407.

[B6] Cryan JR Urban JM (2012) Higher-level phylogeny of the insect order Hemiptera: is Auchenorrhyncha really paraphyletic? Systematic Entomology 37: 7–21. doi: 10.1111/j.1365-3113.2011.00611.x

[B7] Cui Y Xie Q Hua J Dang Kai Zhou J Liu X Wang G Yu Xin Bu W (2013) Phylogenomics of Hemiptera (Insecta: Paraneoptera) based on mitochondrial genomes. Systematic Entomology 38(1): 233–245. doi: 10.1111/j.1365-3113.2012.00660.x

[B8] Frydrychová R Grossmann P Trubač P Vitková M Marec F (2004) Phylogenetic distribution of TTAGG telomeric repeats in insects. Genome 47: 163–178. doi: 10.1139/g03-100 15060613 10.1139/g03-100

[B9] Frydrychová R Marec F (2002) Repeated losses of TTAGG telomere repeats in evolution of beetles (Coleoptera). Genetica 115(2): 179–187. doi: 10.1023/A:1020175912128 12403172 10.1023/a:1020175912128

[B10] Gokhman VE Anokhin BA Kuznetsova VG (2014) Distribution of 18S rDNA sites and absence of the canonical TTAGG insect telomeric repeat in parasitoid Hymenoptera. Genetica 142(4): 317–322. doi: 10.1007/s10709-014-9776-3 24992984 10.1007/s10709-014-9776-3

[B11] Golub NV Kuznetsova VG Rakitov RA (2014) First karyotype data on the family Myerslopiidae (Hemiptera, Auchenorrhyncha, Cicadomorpha). Comparative Cytogenetics 8(4): 293–300. doi: 10.3897/CompCytogen.v8i4.8813 25610543 10.3897/CompCytogen.v8i4.8813 PMC4296716

[B12] Golub NV Golub VB Kuznetsova VG (2015) Variability of 18rDNA loci in four lace bug species (Hemiptera, Tingidae) with the same chromosome number. Comparative Cytogenetics 9(4): 513–522. doi: 10.3897/CompCytogen.v9i4.5376 10.3897/CompCytogen.v9i4.5376 PMC4698567 26753071

[B13] Grozeva S Nokkala S (1996) Chromosomes and their meiotic behavior in two families of the primitive infraorder Dipsocoromorpha (Heteroptera). Hereditas 125: 31–36. doi: 10.1111/j.1601-5223.1996.t01-1-00031.x

[B14] Grozeva S Kuznetsova V Anokhin B (2010) Bed bug cytogenetics: karyotype, sex chromosome system, FISH mapping of 18S rDNA, and male meiosis in *Cimex lectularius* Linnaeus, 1758 (Heteroptera: Cimicidae). Comparative Cytogenetics 4(2): 151–160. doi: 10.3897/compcytogen.v4i2.36

[B15] Grozeva S Kuznetsova V Anokhin B (2011) Karyotypes, male meiosis and comparative FISH mapping of 18S ribosomal DNA and telomeric (TTAGG)n repeat in eight species of true bug (Hemiptera, Heteroptera). Comparative Cytogenetics 5(4): 355–374. doi: 10.3897/CompCytogen.v5i4.2307 24260641 10.3897/CompCytogen.v5i4.2307 PMC3833783

[B16] Grozeva S Anokhin B Kuznetsova VG (2014a) Bed bugs (Hemiptera). In: Sharakhov I (Ed.) Protocols for Cytogenetic Mapping of Arthropod Genomes. CRC press, Taylor & Francis, Boca Raton, 285–326.

[B17] Grozeva S Kuznetsova V Hartung V (2014b) First cytogenetic study of Coleorrhyncha: meiotic complement of *Xenophyes cascus* (Hemiptera: Peloridiidae). European Journal of Entomology 111(2): 303–306. doi: 10.14411/eje.2014.023

[B18] Kuznetsova VG Grozeva SM Nokkala S Nokkala Ch (2011) Cytogenetics of the true bug infraorder Cimicomorpha (Hemiptera, Heteroptera): a review. Zookeys 154: 31–70. doi: 10.3897/zookeys.154.1953 22287915 10.3897/zookeys.154.1953 PMC3238039

[B19] Kuznetsova VG Grozeva S Anokhin B (2012) The first finding of (TTAGG)n telomeric repeat in chromosomes of true bugs (Heteroptera, Belostomatidae). Comparative Cytogenetics 6(4): 341–346. doi: 10.3897/compcytogen.v6i4.4058 24260674 10.3897/CompCytogen.v6i4.4058 PMC3834568

[B20] Kuznetsova VG Maryańska-Nadachowska A Anokhin B Aguin-Pombo D (2015) Evidence for TTAGG telomere repeats and rRNA gene clusters in leafhoppers of the genus *Alebra* (Hemiptera: Auchenorrhyncha: Cicadellidae). European Journal of Entomology 112(2): 207–214. doi: 10.14411/eje.2015.045

[B21] Lachowska-Cierlik D Maryańska-Nadachowska A Kuznetsova V Picker M (2015) First chromosomal study of Mantophasmatodea: Karyotype of *Karoophasma biedouwense* (Austrophasmatidae). European Journal of Entomology.

[B22] Lukhtanov VA Kuznetsova VG (2010) What Genes and Chromosomes Say about the Origin and Evolution of Insects and Other Arthropods. Russian Journal of Genetics 46(9): 1115–1121. doi: 10.1134/S1022795410090279 21061630

[B23] Maryańska-Nadachowska A Kuznetsova V Karamysheva T (2013) Chromosomal location of rDNA clusters and TTAGG telomeric repeats in eight species of the spittlebug genus *Philaenus* (Hemiptera: Auchenorrhyncha: Aphrophoridae). European Journal of Entomology 110(3): 411–418. doi: 10.14411/eje.2013.055

[B24] Nokkala S Nokkala C (1983) Achiasmatic male meiosis in two species of *Saldula* (Saldidae, Hemiptera). Hereditas 99: 131–134. doi: 10.1111/j.1601-5223.1983.tb00737.x 6643081 10.1111/j.1601-5223.1983.tb00737.x

[B25] Nokkala S Nokkala C (1984) The occurrence of the X0 sex chromosome system in *Dictyonota tricornis* (Schr.) (Tingidae, Hemiptera) and its significance for concepts of sex chromosome system evolution in Heteroptera. Hereditas 100: 299–301. doi: 10.1111/j.1601-5223.1984.tb00130.x

[B26] Nokkala S Kuznetsova VG Maryańska-Nadachowska A Nokkala C (2004) Holocentric chromosomes in meiosis. I. Restriction of the number of chiasmata in bivalents. Chromosome Research 12: 733–739. doi: 10.1023/B:CHRO.0000045797.74375.70 15505408 10.1023/B:CHRO.0000045797.74375.70

[B27] Ouvrard D Campbell BC Bourgoin T Chan KL (2000) 18S rRNA secondary structure and phylogenetic position of Peloridiidae (Insecta, Hemiptera). Molecular Phylogenetics and Evolution 16(3): 403–417. doi: 10.1006/mpev.2000.0797 10991793 10.1006/mpev.2000.0797

[B28] Papeschi AG Bressa MJ (2006) Evolutionary cytogenetics in Heteroptera. Journal of Biological Research 5: 3–21. doi: 10.1186/jbiol30

[B29] Popov YuA Shcherbakov DE (1996) Origin and evolution of the Coleorrhyncha as shown by the fossil record. In: Schaefer CW (Ed.) Studies on Hemipteran Phylogeny. Entomological Society of America, Lanham, MD, USA, 9–30.

[B30] Sahara K Marec F Traut W (1999) TTAGG telomeric repeats in chromosomes of some insects and other arthropods. Chromosome Research 7(6): 449–460. doi: 10.1023/A:1009297729547 10560968 10.1023/a:1009297729547

[B31] Schlee D (1969) Morphologie und Symbiose; ihre Beweiskraft fur die Verwandtschaftsbeziehungen der Coleorrhyncha. Stuttgarter Beiträge zur Naturkunde 210: 1–27.

[B32] Štys P Kerzhner I (1975) The rank and nomenclature of higher taxa in recent Heteroptera. Acta Entomologica Bohemoslovaca 72(2): 65–79.

[B33] Traut W Szczepanowski M Vitková M Opitz C Marec F Zrzavy J (2007) The telomere repeat motif of basal Metazoa. Chromosome Research 15(3): 371–382. doi: 10.1007/s10577-007-1132-3 17385051 10.1007/s10577-007-1132-3

[B34] Ueshima N (1979) Hemiptera II: Heteroptera. In: John B (Ed.) Animal Cytogenetics. 3. Insecta 6. Gebrüder Bornträger, Berlin, Stuttgart, 113 pp.

[B35] Vershinina A Anokhin B Lukhtanov V (2015) Ribosomal DNA clusters and telomeric (TTAGG)*_n_* repeats in blue butterflies (Lepidoptera, Lycaenidae) with low and high chromosome numbers. Comparative Cytogenetics 9(2): 161–171. doi: 10.3897/CompCytogen.v9i2.4715 26140159 10.3897/CompCytogen.v9i2.4715 PMC4488964

[B36] Vitková M Kral J Traut W Zrzavy J Marec F (2005) The evolutionary origin of insect telomeric repeats, (TTAGG)*_n_*. Chromosome Research 13: 145–156. doi: 10.1007/s10577-005-7721-0 15861304 10.1007/s10577-005-7721-0

[B37] Wheeler WC Schuh RT Bang R (1993) Cladistic relationships among higher groups of Heteroptera: congruence between morphological and molecular data sets. Entomologica Scandinavica 24: 121–137. doi: 10.1163/187631293X00235

[B38] White MJD (1973) Animal cytogenetics and evolution. Cambridge, 961 pp.

[B39] Wootton R (1965) Evidence for tracheal capture in early Heteroptera. Proceedings of the XII International Congress of Entomology, London, 8-16 July, 1964 London, 65–67.

